# Droplet Microfluidic Hydrogen/Deuterium Exchange for Investigating Protein Dynamics with Millisecond Precision

**DOI:** 10.1021/acs.analchem.6c02766

**Published:** 2026-09-28

**Authors:** Dietmar Hammerschmid, Jakub Sys, Alistair Bailey, Simon I. R. Lane, Max Saito, Theo Hornsey, Niall Hanrahan, Andy van Hateren, Howard Broughton, Alfonso Espada, Eamonn Reading, Jonathan West

**Affiliations:** † School of Biological Sciences, Faculty of Environmental and Life Sciences, 7423 University of Southampton, Southampton SO17 1BJ, U.K.; ‡ Cancer Sciences, Faculty of Medicine, University of Southampton, Southampton SO17 1BJ, U.K.; § School of Chemistry, Faculty of Engineering and Physical Sciences, University of Southampton, Southampton SO17 1BJ, U.K.; ∥ Institute for Life Sciences, University of Southampton, Southampton SO17 1BJ, U.K.; ⊥ Centro de Investigación Lilly, SA, Avenida de la Industria 30, Alcobendas 28108, Spain

## Abstract

Hydrogen/Deuterium eXchange (HDX) methods for studying protein dynamics would benefit from millisecond-scale incubations to probe intrinsically disordered proteins, highly dynamic regions, and conformation changes. Here, we investigate droplet microfluidics for rapid mixing to trigger D_2_O labeling, uniform incubations, and rapid droplet merging for acid quenching in advance of mass spectrometry. A surfactant-free merging approach combining expansion elements for synchronized droplet collision proved robust. The high diffusive flux of D_2_O and protons enables microsecond mixing to trigger and arrest D_2_O labeling, respectively, affording the possibility of single millisecond incubations. Droplet HDX processors were used to measure the fast uptake characteristics of a model peptide. Forward exchange measurements demonstrate D_2_O labeling to be the rate-limiting step, in essence defining 10 milliseconds as the minimum practical incubation time for proteins at room temperature, pD 7.4. With the ability to access millisecond time scales, the fast dynamics of calmodulin, a model of calcium-triggered allostery with rapid conformational switching, was investigated. At 10 milliseconds, we could observe significant deuterium uptake within the well-defined EF-hand Ca^2+^ binding motifs. These findings demonstrate that millisecond HDX enabled by droplet microfluidics allows areas of heightened plasticity to be detected within a stably folded protein.

## Graphical abstract

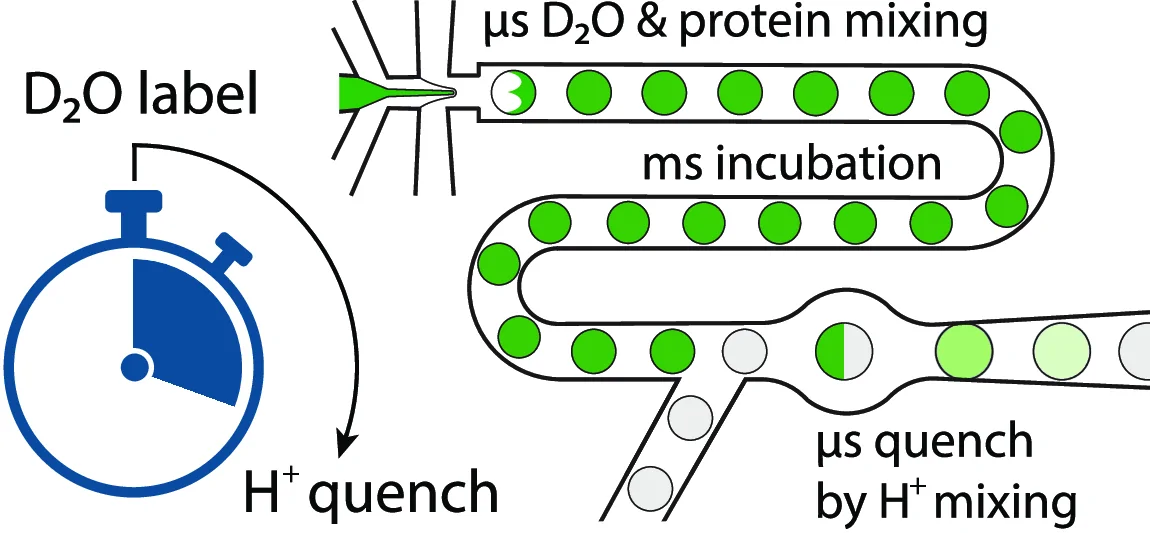

## Introduction

Protein motion enables function, with celebrated examples being the oar-like stroke of myosin during muscle contraction, ATP synthase rotation, and the valving of ligand-gated ion channels. We have a vast library of high-resolution protein structures, largely determined using crystallography, yet we know relatively little about protein motion. This knowledge gap represents an enormous arena for discovery and opportunity to advance biotechnology, inform the development of new drugs (e.g. shifting conformational equilibria towards health), and the design of synthetic proteins. While time-resolved crystallography is maturing and delivers atomic resolution,^1^ routine access to beamlines with appropriate infrastructure and the pre-requisite for large quantities of well-formed protein crystals limit our ability to broadly understand protein dynamics. Instead, time-resolved cryo-electron microscopy (cryo-EM) is emerging^2–6^ and complements a battery of methods providing insight into protein motion. Of these, a technique called Hydrogen/Deuterium eXchange (HDX) has its place. Here, labile hydrogen atoms on the amide backbone are replaced by deuterium atoms from the surrounding solvent. These can be measured by nuclear magnetic resonance (NMR) or mass spectrometry (MS) and reflect backbone malleability and accessibility from which protein structure and dynamics can be inferred. HDX-MS is gaining popularity across academic and pharmaceutical sectors owing to improved throughput, reduced sample consumption, and capacity for analyzing complex samples.^7,8^

The HDX experiment involves (i) mixing protein with D_2_O (deuterium), (ii) defined incubation periods, and then (iii) mixing with an acidic quench buffer to preserve D_2_O incorporated into the amide backbone for (iv) bottom-up peptide level analysis, typically by mass spectrometry. Protein structure governs the hydrogen-bonding network stability and solvent accessibility, which in turn dictate the rate of D_2_O uptake. Exchange occurs at all time scales, with the cumulative extent reflecting structural dynamics. At stably structured regions, the breakage of backbone hydrogen bonds is infrequent, requiring long periods for the HDX reaction to introduce D_2_O. In contrast, unstructured regions are exposed and show low level backbone stabilization allowing the HDX reaction to introduce D_2_O within short time scales. Conventional HDX workflows are limited to multisecond incubations, thereby overlooking important protein structural features such as those involved in binding and sensing. Crucially, millisecond time scales also correspond to protein folding,^9^ allosteric regulation,^10^ and intrinsic disordering.^11^ Millisecond HDX D_2_O labeling times also make possible time-resolved non-equilibrium measurements involving pulsed (triggered) experiments, with the enticing possibility to observe motions arising from biomolecular interactions such as ligand binding and substrate catalysis.^7,12,13^

To access single second and millisecond time scales, manual or robotic processing in wells is replaced with continuous flow approaches within capillaries or microchannels. Examples include quenched flow-like arrangements,^9,14,15^ or laminar flow systems with mixing within capillaries,^16–18^ the convoluted paths within porous monoliths,^19^ or by switching between different chemical streamlines.^20,21^ Recently, turbulent transport in capillaries is used for rapid mixing in an online, fully automated HDX-MS platform.^11,22^ Within these examples, multi-fold challenges and associated drawbacks emerge: the requirement for rapid mixing (substantially faster than incubations), uniform incubation times, and reliability coupled with simple and low-cost instrumentation to support wider access for the HDX community. To date, the shortest achievable incubation with these methods is 50 milliseconds,^16,22^ providing motivation to explore methods for shorter incubations which necessarily require exceptionally fast mixing.

In this study, we turn our attention to droplet microfluidics, famed for rapid mixing enabled by circulations within droplets.^23,24^ In addition, single-file droplet transport by channel confinement bypasses the Taylor-Aris dispersion^25,26^ problem encountered with ordinary microfluidics, to achieve extremely uniform incubations.^23,24,27,28^ The remaining challenge is the need to rapidly introduce and mix the quench buffer with the protein/D_2_O droplets at a precisely defined time. This can be achieved through droplet merging. To favor user uptake, we have considered passive methods involving straightforward and inexpensive fabrication, reduced instrumentation, and reduced complexity. In this study, we explore 3 approaches for rapidly introducing quench buffer to high-velocity D_2_O/protein droplets and identify a surfactant-free droplet collision approach as being the most robust. We demonstrate microsecond mixing, facilitating incubations as short as 1 millisecond. The ability to access such short time scales with high temporal precision in an inexpensive format requiring minimal sample will be of interest for the community to study highly dynamic proteins, non-equilibrium measurements, or dynamics underpinning catalysis. Using this improved temporal resolution, we experimentally explore the limits of D_2_O labeling and compare results with theory. To test drive droplet microfluidic HDX for probing highly dynamic regions, we investigated calmodulin, a model of calcium-mediated allostery.

## Materials and Methods

### Microfluidic Design

Devices consist of a droplet generation junction for adding D_2_O to protein, then an incubation channel (with length and velocity defining incubation times) which concludes by joining with a channel for introducing quench buffer to arrest labelling (Figure S1). The “chop and exchange” device has adjoining channels downstream for the addition of perfluoro-2-octanol, which exchanges with the surfactant at the droplet interface to cause droplets in contact to merge (Figure 1A). The “drag and merge” device simply relies on a carrier fluoro-oil without surfactant, with protein/D_2_O droplets contacting a stream of quench buffer to produce daughter droplets containing near-equivalent volumes of both (Figure 1B). The “collide and merge” device employs two droplet generation junctions, one for producing protein/D_2_O droplets and the other for producing quench droplets. Droplets are again produced using fluoro-oil without surfactant and merge upon collision in a channel expansion-contraction element where lateral oil drainage ensures droplets contact one another and merge (Figures S2A, 1C and 2B). For short, 1 millisecond incubations, smaller and higher velocity droplets are required for mixing in the order of 100 μs. These small and fast droplets require steering towards the channel center to ensure droplets contact one another for reliable merging and mixing. This was achieved using pillar structures similar to those described by Niu et al.^29^ (Figures S2B and 2D) which facilitate lateral oil drainage, while positioning droplets at the channel center.

**Figure 1:**
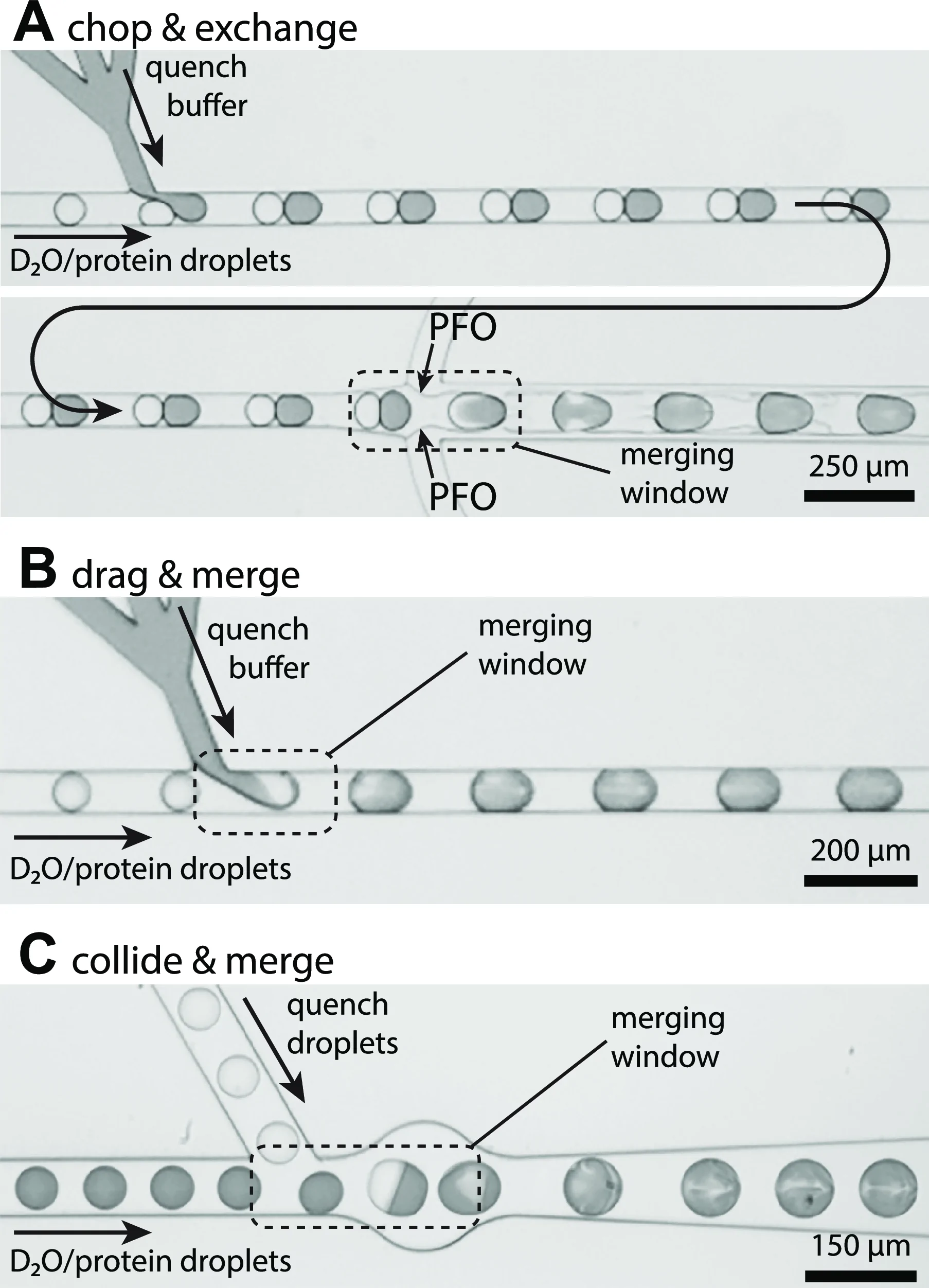
Droplet merging strategies. (A) “Chop and exchange”: The momentum of the first, protein/D_2_O, droplet chops off a droplet from the adjoining quench buffer channel. Perfluoro-1-octanol (PFO) is introduced downstream, destabilizing droplet interfaces to cause merging. (B) “Drag and merge”: Without surfactants, viscous drag between the protein/D_2_O droplet and quench buffer produces a daughter droplet with equal volumes of protein/D_2_O and quench buffer. (C) “Collide and merge”: Protein/D_2_O and quench buffer droplets are generated independently without surfactant, and merge upon collision. Channel expansion ensures all droplets merge.

**Figure 2:**
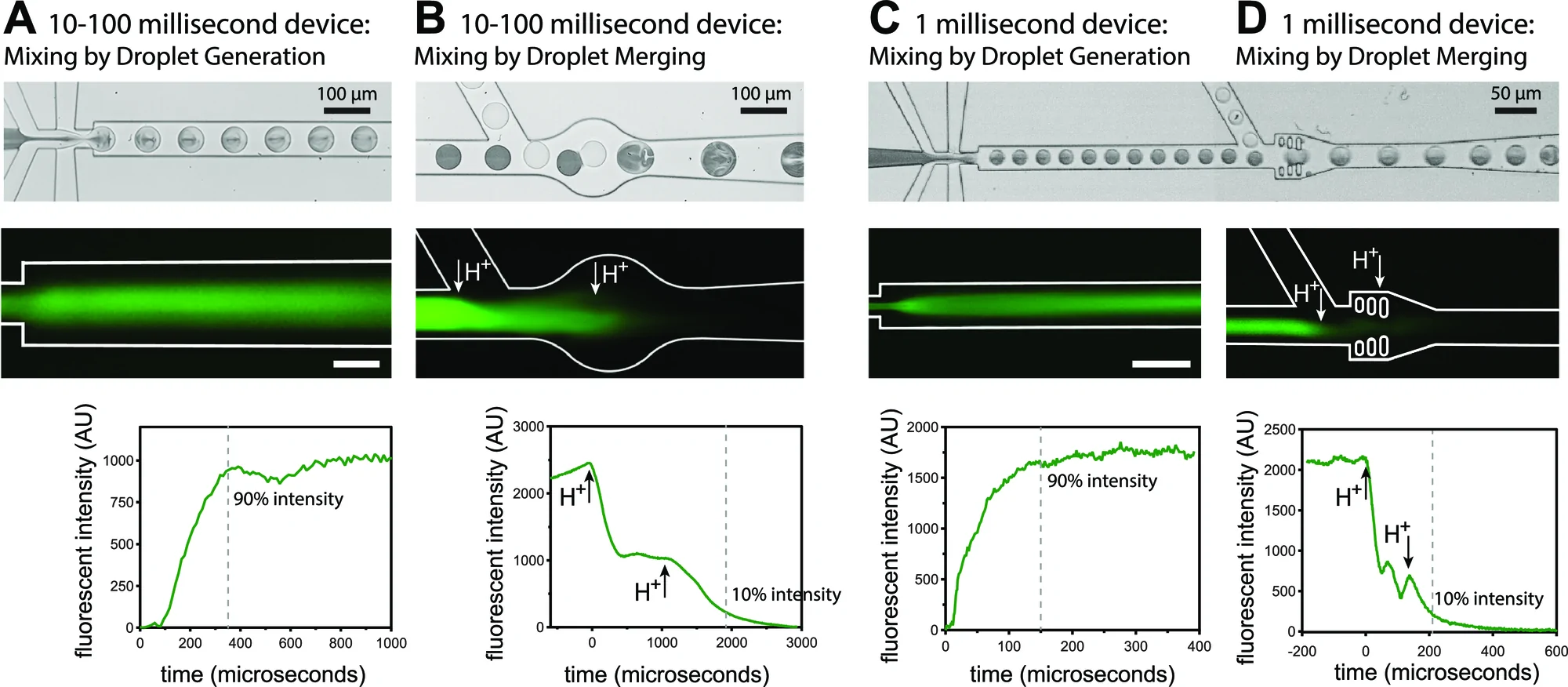
Mixing time analysis. (A) Droplet generation in the 10 and 100 millisecond incubation devices. Acidified fluorescein on-switching with hydroxide was used to estimate a mixing time of ∼350 μs. (B) Droplets collide as channels converge or collide in the channel expansion region to produce two mixing times estimated using fluorescein off-switching with HCl to be ∼350 μs and ∼1 millisecond, respectively. (C) Faster mixing occurs in the 1 millisecond droplet generation device, with a fluorescein on-switching time of ∼150 μs. (D) Droplets collide as channels converge and in the oil drainage element. The collective fluorescein off-switching time is ∼200 μs. Attaining 90% fluorescent intensity was used to estimate the D_2_O mixing time, and reduction to 10% was used to estimate the acid mixing time. Fluorescent image scale bars represent travel durations of 25 μs (A) and 100 μs (C).

### Microfabrication and Assembly

Droplet microfluidic HDX devices were fabricated in poly­(dimethylsiloxane) (PDMS, Sylgard 184) by standard soft lithography using SU-8 on silicon masters. Devices for 10- and 100-millisecond incubations were fabricated with a height of 41 μm, and with a channel width of 75 μm and a droplet junction width of 40 μm. The “collide and merge” device has a channel expansion element with a 600 pL half-volume (Figure S2A, SI CAD). The “collide and merge” device for 1 millisecond incubations was fabricated to a height of 14 μm, and with a channel width of 30 μm, a droplet junction width of 15 μm, and with a 21 pL-volume cavity with oil-drainage pillars (Figure S2B, SI CAD). PDMS was cured on the SU-8 wafer at 60 °C for 2 h and a counter-molding strategy was used to produce polyurethane masters.^30^ These masters were used for replicating devices in PDMS, again with curing at 60 °C for 2 h. Tubing inlet ports were introduced using 1 mm-diameter biopsy punches (Miltex, Williams Medical Supplies Ltd). A 1.5 mm-diameter biopsy punch was used for the exit port. A 30 s oxygen plasma treatment (Femto, Diener Electronic) was used to bond microfluidic devices to a glass microscope slide. Subsequently, channel surfaces were functionalized by injecting a 5 μL volume of 1% (v/v) trichloro­(1*H*,1*H*,2*H*,2*H*-perfluorooctyl) silane (Merck) in HFE-7500 (3M Novec).

### Microfluidic Operation

Syringe pumps (Fusion 100, Chemyx) were used to deliver reagents from gas tight syringes (SGE) to the droplet microfluidic HDX device. Syringes were fitted with 25-gauge (OD 0.5 mm) needles for interfacing with polytetrafluoroethylene (PTFE, Bohlender, Merck) tubing with an ID of 0.5 mm. The 1 mm OD of the tubing allows secure attachment, by insertion, into the device ports. For retrieving sample from the device, 1.6 mm OD PTFE tubing (Cole-Palmer) was used which fits securely in the 1.5 mm-diameter exit port. This tubing has a 0.3 mm ID for rapid sample delivery (to reduce forward and back exchange) to a 0.5 mL microcentrifuge tube placed in a 3D printed (UltiMaker Cura) dry ice bath designed to sit on the microscope stage in close proximity to the microfluidic HDX device. Tubing was inserted through a hole in the microcentrifuge tube lid prepared using a biopsy punch. Tubing should not be inserted to the bottom of the microcentrifuge tube to avoid sample freezing within the tubing and blocking flow. Once collected, volumes were flash frozen in liquid nitrogen, then stored at −80 °C in advance of mass spectrometry. Droplets coalesce in the collection tube into a single aqueous phase above the denser HFE-7500 layer (1.61 g/cm^3^). Once thawed, the sample was readily retrieved using a pipette. Oil contamination, impacting digestion, separation, and ion suppression was not observed. Droplet generation and merging was monitored using a high-speed camera (Phantom Miro M310, Vision Research) mounted on an inverted microscope (Olympus, CKX53). A 9:1 ratio of D_2_O to protein volume was used for labelling and a 1:1 ratio of D_2_O/protein to quench buffer volume was used to quench labelling. Liquids and flow rates for the different microfluidic HDX methods are documented in Table 1.

**Table 1:** Microfluidic HDX methods, incubation channel dimensions, liquids, and flow rates^a^

microfluidic method	channel height (μm)	channel width (μm)	fluoro-oil flow rate (μL/min)	protein flow rate (μL/min)	D_2_O flow rate (μL/min)	quench flow rate (μL/min)	other flow rates (μL/min)
chop & exchange	41	75	25 (1% QX200)	1	9	10	8 (PFO)
drag & merge	41	75	25 (HFE-7500)	1	9	10	-
collide & merge (10–100 ms)	41	75	20 (HFE-7500)	1	9	10	20 (HFE-7500)
collide & merge (1 ms)	14	30	7.5 (HFE-7500)	0.25	2.25	2.5	7.5 (HFE-7500)

a The carrier phase for droplet generation was 1% QX200 (BioRAD, providing surfactant) in HFE-7500 (3M Novec) or just HFE-7500. The surfactant in QX200 was exchanged using neat perfluoro-1-octanol (PFO, Merck). A 100 μL aqueous volume was required for MS measurements, involving 5 min processing times for the 10 and 100 ms incubation devices and 20 min processing times for the 1 ms incubation device. The processing time is dictated by the volume requirement of the sample loop and the dead volume associated with MS injection. Reduced processing times can be achieved by using higher concentration protein inputs, followed by dilution during sample retrieval to attain the desired volume.

### Mixing Analysis

A red dye, sulfanilic acid azochromotrop (SAA, Merck, λ_max_ 505–510 nm, 10 mM), was used to observe mixing during droplet generation and droplet merging. Fluorescein pH-switching^31^ was used to estimate protein mixing times with D_2_O during droplet generation and protein/D_2_O mixing times with acidic quench buffer during droplet merging: Addition of 1M hydroxide to acidified 10 mM fluorescein neutralizes the solution and restores fluorescent emission (λ_max_ 517 nm), whereas addition of 10 mM HCl (pH 2.0) to 10 mM fluorescein switches off fluorescence. Here, the hydroxide (*M*_wt_ 17 Da) closely matches the diffusivity of D_2_O (*M*_wt_ 20 Da) and HCl addition mimics the quenching reaction. A sensitive camera (Fusion, Hamamatsu) mounted on an inverted microscope (CKX41, Olympus) was used for fluorescent imaging with peak excitation at 490 nm (pE-100 CoolLED) and emission collection at 515 ± 10 nm. A 200 ms exposure was used to collect fluorescent emission from large numbers of droplets in a single frame (∼300 large droplets (∼110 pL) and ∼3000 small droplets (∼3 pL)) to provide ensemble measurements. The plot profile function in Fiji was used to quantify fluorescence development during droplet generation and fluorescence reduction following droplet merging. Position data was converted to time using channel location-determined velocities (e.g., droplet acceleration where streams converge and velocity changes within the expansion-contraction element).

### Millisecond Labelling of Peptides

Peptide AEAKQNLGNAKQK (Synpeptide) was solubilized in equilibration buffer (20 mM HEPES, pH 7.4) at a concentration of 1 μM. D_2_O labelling was performed for 10 and 100 milliseconds as described in the microfluidic operation section using a 9:1 ratio of labelling buffer (20 mM HEPES in D_2_O, pD 7.4) to peptide volume and a 1:1 ratio of D_2_O/protein to quench buffer (100 mM NaH_2_PO_4_, pH 2.3) volume. Samples were collected for 5 min on dry ice, flash frozen in liquid N_2_, and stored at −80 °C in readiness for mass spectrometry. A control sample was prepared by 10-fold dilution in equilibration buffer before mixing 1:1 with quench buffer.

### Forward Exchange Measurements

Back exchange, the loss of deuterium label from the amide backbone, and forward exchange, gain of deuterium label after quench, can occur with delays between quench and analysis. With short labelling times forward exchange, although slow, can increase deuterium labelling. To measure forward exchange, we added D_2_O at the quench step,^32^ not the initial step. Uptake was quantified at peptide level after calmodulin pre-digestion: Calmodulin (CaM, from bovine testes Cat No. P1431-1MG, Merck) was solubilized at 1 mg/mL in equilibration buffer (20 mM HEPES, pH 7.4) and mixed 1:1 with quench buffer (100 mM NaH_2_PO_4_, pH 2.3) to a final concentration of 2 and 20 μM for forward exchange and standard labelling, respectively. Quenched CaM digestion was performed on an ACQUITY UPLC System with HDX Technology (Waters, Wilmslow, UK). The HDX manager of the system was equipped with a self-packed guard column (2.0 mm ID × 2 cm unpacked; Part No. C–130B) containing immobilized pepsin agarose resin (Cat No. 20343, Thermo Fisher). Samples were injected into the HDX manager and run with solvent A (0.23% formic acid, pH 2.5) over the self-packed pepsin column at 200 μL/min. Digested CaM was collected for 1 min at the column outlet, flash frozen in liquid N_2_, and freeze-dried for 16 h overnight. For mass spectrometry, samples were resuspended in 100 μL equilibration buffer and sonicated for 15 min. Standard and forward exchange labelling were then performed on the respective droplet microfluidic HDX device (1, 10, and 100 microseconds). Standard labelling was performed as described in the microfluidic operation section using a 9:1 ratio of labelling buffer (20 mM HEPES in D_2_O, pD 7.4) to protein volume and a 1:1 ratio of D_2_O/protein to quench buffer (100 mM NaH_2_PO_4_, pH 2.3) volume. For forward exchange labelling, quench buffer was prepared in 90% D_2_O (100 mM NaH_2_PO_4_, pD 2.3) to reach an equal deuterium content in the final sample. Labelling experiments were repeated at pD 9.4 using 20 mM sodium carbonate-bicarbonate D_2_O labelling buffer (pD 9.4) and 1.25% formic acid quench buffer. The flow rates for the 10 and 100 millisecond incubations require 5 min to collect 100 μL aqueous volume, whereas the lower flow rate for the 1 millisecond incubation required 20 min to collect this volume. Using different tubing lengths, transport time from device exit to collection on dry ice was standardized to 20 s. Once collected, samples were flash frozen in liquid N_2_ and stored at −80 °C in readiness for mass spectrometry.

### Investigating Calmodulin Dynamics

Calmodulin (CaM, from bovine testes Cat No. P1431-1MG, Merck) was solubilized at 1 mg/mL in equilibration buffer (20 mM HEPES, pH 7.4). To prepare Ca^2+^-free CaM samples, 1 mM ethylenediaminetetraacetic acid (EDTA) was added to the CaM and incubated for 16 h at room temperature. CaM dynamics were investigated with and without Ca^2+^ to characterize protein states at millisecond to second time scales (10 and 100 millisecond microfluidic, and 10, 100, and 1000 s manual labelling). Ca^2+^-bound CaM was prepared by adding 1 mM CaCl_2_ and incubation at room temperature (22 °C) for at least 3 h prior to deuterium labelling. For CaM without Ca^2+^, all buffers were also equilibrated to room temperature. After equilibration, the exchange reaction was initiated by 10-fold dilution into deuterated labelling buffer (20 mM HEPES, pH_read_ 7.0), yielding a 90% deuterium content in the final reaction mixture. Ca^2+^-bound CaM was deuterated with Ca^2+^-labelling buffer (20 mM HEPES, 1 mM CaCl_2_, pH_read_ 7.0). To deuterate Ca^2+^-free CaM samples, Ca^2+^-free labelling buffer (20 mM HEPES, pH_read_ 7.0) was applied. Reactions were quenched by 1:1 dilution with quench buffer (100 mM NaH_2_PO_4_, pH 2.3). Samples incubated for millisecond time scales (10 and 100 milliseconds) were processed as described above (microfluidic operation), with the exception of 8 s transport times from device to dry ice. 10, 100, and 1000 s labelling was performed manually by introducing labelling and then quench buffer to the aliquoted samples. All protein states and labelling time points were performed in three technical replicates on two occasions. Samples processed by microfluidic and manual methods were stored at −80 °C for a maximum of 24 h prior to mass spectrometry. Deuterium uptake values were corrected using experimentally determined maximum deuteration (Dmax) values. Fully deuterated control samples were prepared and analyzed in triplicate according to the protocol described by Peterle et al.,^33^ involving incubation of the protein under denaturing conditions at elevated temperature in a D_2_O-based buffer to maximize deuterium incorporation. *D*_max_ values were used to correct for back exchange and normalize the observed deuterium uptake measured for each peptide at each labelling time point.

Of note, when performing similar Ca^2+^-stripping procedures on bovine CaM recombinantly expressed in *Escherichia coli* (sourced from Cat No. C4874-1MG, Merck), insignificant differences between intended apo and holo states were observed within our HDX-MS analysis (data included in data repository PXD063880). With the apo state presenting as the holo state, this suggests that cation stripping is more challenging from this construct and supports the value in exploring proteins sourced from different cell types (or species).^34^

### Mass Spectrometry Measurements

Measurements were performed on an ACQUITY UPLC M-Class System with HDX Technology (Waters, Wilmslow, UK) directly coupled to a Xevo G2-XS QToF mass spectrometer (Waters, Wilmslow, UK). Frozen samples were quickly thawed, and injected into the HDX manager. For millisecond sample labelling, only the upper aqueous phase was injected. Proteins were digested on-line using a self-packed pepsin (immobilized pepsin agarose resin, Cat No. 20343, Thermo Fisher) column (2.0 mm ID x 2 cm unpacked; Part No. C–130B) at 15 °C, and resulting peptides were trapped/washed on an Acquity UPLC BEH C18 VanGuard pre-column (1.7 μm, 2.1 mm × 5 mm, Waters, Wilmslow, UK) for 3 min at 200 μL/min. The self-packed pepsin column was replaced by a union for samples containing pre-digested calmodulin (i.e., samples for comparing forward exchange with standard labelling). Peptides were eluted and separated on an Acquity UPLC BEH C18 analytical column (1.7 μm, 1.0 mm × 100 mm, Waters, Wilmslow, UK) with a linear gradient over 7.5 min from 8 to 35% solvent B (0.23% formic acid in acetonitrile) at a flow rate of 40 μL/min and at 0 °C. To prevent peptide carryover, the pepsin column was washed twice during the linear gradient using a pepsin wash solution (1.6 M guanidine-HCl, 4% acetonitrile, and 0.8% formic acid, pH 2.5). Further, chromatographic columns were washed after each sample run by applying a sawtooth gradient.

### HDX-MS Data Analysis

For peptide identification, undeuterated calmodulin was injected into the system applying the same settings for protein digestion and chromatographic separation. Peptides were measured in positive ion mode and MS^E^ analysis was applied with a ramped collision energy from 20 to 45 V. Sodium iodide and leucine enkephalin were used for calibration and mass accuracy correction, respectively. Raw data from MS^E^ runs were analyzed with ProteinLynx Global Server (PLGS) 3.0 (Waters, Wilmslow, UK). Identified peptides were loaded into DynamX 3.0 (Waters, Wilmslow, UK) and filtered as follows: minimum intensity: 3000, sequence length: 0–25, minimum products per amino acid: 0.1, minimum consecutive products: 0, minimum score: 8, maximum MH + error: 10 ppm, file threshold: 2 out of 3 and 3 out of 4 measurements for millisecond and manual data sets, retention time RSD: 5%.^35^ Peptides were manually curated to exclude MS traces of poor quality or false identifications. Subsequently, raw data from deuterated samples (with and without Ca^2+^) were loaded to calculate the deuterium uptake at peptide level. To determine statistically significant differences in deuterium uptake between two protein states, only peptides present in both data sets were filtered and a global 99% confidence threshold was calculated based on a previous approach^36^ including recently proposed corrections.^37^ The mass spectrometry proteomics data have been deposited to the ProteomeXchange Consortium via the PRIDE^38^ partner repository with the dataset identifiers PXD063880 and PXD077479. Details of the calmodulin experiment are recorded in the Table S1: HDX Summary.

## Results and Discussion

### Droplet Merging Strategies

Achieving millisecond HDX incubations requires rapid mixing of protein with D_2_O, defined incubations and subsequently rapid mixing with acid quench buffer. While droplet microfluidics enables rapid mixing and precisely defined incubations, the task of reliable droplet merging at the correct position (i.e., time) and at high velocity for rapid mixing required investigation. Several on-the-fly droplet-merging methods have been developed. Surface free energy patterning within microchannels presents a hydrophilic stripe for droplet trapping and merging.^39,40^ Alternatively, electrocoalescence reliably achieves merging at a defined location.^41–45^ In pursuit of straightforward, low-cost fabrication and instrumentation to favor end-user adoption, we instead chose to investigate passive methods. Initial experiments used depleted surfactant to allow the aqueous compartments of two droplets to contact one another and coalesce.^46,47^ However, reliability issues emerged and the merging position varied, resulting in variable incubations. We next investigated other passive droplet merging techniques and their suitability for fast HDX.

We explored the injection of quench buffer from a side channel into passing droplets that were generated upstream.^48,49^ Using low concentration surfactant (1% QX200 in HFE-7500), droplet momentum and interfacial tension act like a chopper, producing a second droplet of quench buffer which is synchronized with the D_2_O droplet. Merging at a defined downstream location is achieved by the introduction of perfluoro-1-octanol (PFO), a weak surfactant which exchanges with the QX200 surfactant, momentarily producing surfactant-free pin holes at the interface between droplets to trigger merging (“chop and exchange” method, Figure 1A and Video S1). However, the process was not reliable, proving to be sensitive to subtle physical and chemical variations in reagents^29^ which either prevented chopping or merging. We next considered a more straightforward approach involving the generation of surfactant-free D_2_O droplets with the injection of quench buffer into passing droplets. Here, the viscous interaction between D_2_O droplets and quench buffer caused an equal volume of quench buffer to be dragged from the input stream and, in the absence of surfactant, instantly merged with the protein/D_2_O droplet (“drag and merge” method, Figure 1B–Video S2). Again, this synchronizes protein/D_2_O and quench buffer inputs, with merging at a defined position. Nevertheless, the method was prone to streaming jets of quench buffer, losing positional and associated temporal control, as well as the small volume circulations necessary for rapid mixing.

With the emphasis on reliability and speed, we opted to independently generate surfactant-free protein/D_2_O and quench buffer droplets at matched frequencies for pairing.^27^ However, droplets are not synchronized, either merging as channels converge or missing one another to form an alternating train of droplets. To address this, we installed a fail-safe in the form of a channel expansion-contraction element.^50–52^ On entering the expansion channel region, the droplets decelerate and remain at the channel center ensuring droplets contact one another and merge in microseconds (“collide and merge” method, Figure 1C and Video S3). The channel subsequently contracts, allowing merged droplet pairs to accelerate for faster mixing. Merging occurs in the expanding half of the cavity, with the simple design rule of the half-cavity volume (600 pL) being ≥4 and ≤6 droplet volumes (for a 2:1 fluoro-oil:aqueous flow ratio). This surfactant-free collide and merge strategy proved highly reliable, ensuring that all protein/D_2_O droplets merge with quench buffer either as channels converge or immediately downstream in the expansion-contraction element. Settling on the collide and merge strategy, we designed HDX microfluidic circuits for 10 and 100 millsecond incubations. These circuits produced merging efficiencies of 100% (10 ms; *N* = 1466/1466) and 99% (100 ms; *N* = 1287/1296). Downstream from the expansion-contraction element the channel widens, further coalescing droplets into larger droplets. Consequently, all protein molecules combine with quench buffer, with only a small fraction of molecules experiencing extended incubations of <2 milliseconds. The 10 and 100 millisecond incubation times are themselves highly reproducible (CV <1.6%) and result from ordered droplet transport in the confines of the incubation channel producing uniform velocities.

For shorter, 1 millisecond incubations, faster mixing is required and was achieved by channel miniaturization to produce smaller droplets (3 pL, see Table 1). Such small droplets provide shorter diffusion paths and also involve higher velocity transport (∼795 mm/s vs ∼325 mm/s for ∼110 pL droplets) which enhances convection. At these scales, a higher 3:1 fluoro-oil:aqueous flow ratio was required for reliable droplet generation, with the expansion cavity volume being ≥5 and ≤8 droplet volumes. In addition, the expansion-contraction element required the inclusion of drainage pillars similar to the design of Niu et al.,^29^ which act to confine droplets to the channel center to ensure contact and merging. A 97%, merging efficiency was recorded (*N* = 1711/1763), with the remaining droplets merging downstream within 300 μs. Incubation times within the 1 millisecond channel were also reproducible (CV 1.4%).

### Microsecond Mixing

A red dye was used to gain a first understanding of mixing for triggering D_2_O labelling and mixing following droplet merging for acid quenching (Figures 1 and 2). Previous experiments using 126 pL and 39 pL droplets with associated velocities of 60 mm/s to 300 mm/s, produced mixing times of 6 milliseconds and 2 milliseconds, respectively.^30^ However, the colourimetric dye approach neglects the high diffusivity (1.7 × 10^–5^ cm^2^/s^53^) and flux^54^ of D_2_O (55.5 Molar) for triggering labelling and the extremely high diffusivity of protons (9.3 × 10^–5^ cm^2^/s^55,56^ ) during quenching. To approximate these characteristics, we used fluorescein emission (λ_max_ 517 nm) modulation by pH-switching.^31^ At pH 3 or below, relative fluorescence is near zero, but as the pH increases, so does the fluorescence. To mimic D_2_O mixing, we combined a stream of 1 M hydroxide (*M*_wt_ 17 Da versus D_2_O *M*_wt_ 20 Da) with acidified 10 mM fluorescein (pH 3) during droplet generation and imaged fluorescence development along the channel. To mimic acid quenching during droplet merging and mixing, a 10 mM HCl solution (pH 2) was combined with 10 mM fluorescein at pH 7.0. Fluorescence reduction along the channel was used to measure mixing and reaction times for the overall quench process.

When 1 M hydroxide is used to approximate D_2_O diffusion and flux, mixing times during droplet generation and transport are markedly reduced compared to observations with red dye. In the 10 and 100 millisecond incubation devices, mixing times are ∼350 μs (Figure 2A). Two positions emerge for droplet merging, first where channels converge and secondly in the expansion channel region (Figure S2A). Using fluorescein off-switching with HCl, the first merging event has a mixing time of ∼350 μs, with the latter merging event being extended to ∼1 millisecond as a result of reduced velocity in the expansion-contraction element (Figure 2B). These mixing times are suitably fast to plausibly define 10 and 100 millisecond incubations (i.e., substantially shorter than the incubation times).

Shorter incubations required faster mixing achieved by droplet miniaturization. Mixing during droplet generation in the 1 millisecond incubation device was decreased to ∼150 μs (Figure 2C). Merging occurred at channel convergence and within the oil-drainage pillars, overall producing a mixing time of 200 μs (Figure 2D). With ensembled measurements involving ∼3000 droplets for each location, the mixing times of individual droplet pairs cannot be imaged. However, the sharp drop in fluorescence when channels first converge indicates <50 μs mixing times and ∼100 μs when transported within the lower velocity pillar structure (Figures 2D and S2B). These are suitably fast for single millisecond incubations. Incubations shorter than 1 millisecond could be accessed using smaller and still faster droplets but will be hampered by difficulties of synchronizing smaller droplet pairs at a given location and for sufficient time for oil drainage to allow droplets to contact and merge. Indeed, 1 millisecond incubations may likely prove the limit for reliable droplet processing.

### Millisecond Calibration with Peptides

With precision millisecond incubations afforded by droplet microfluidic HDX, we went on to test millisecond D_2_O labeling of peptides. Ordinarily, peptides do not have secondary structure, providing random-coil characteristics that allow for comparison of the measured D_2_O uptake with theoretical exchange. Using droplet microfluidics, we labeled a peptide (AEAKQNLGNAKQK) for 10 and 100 milliseconds. D_2_O uptake is +2.26 Da with 10 millisecond incubations and increases to +5.44 Da with a 100 millisecond incubation (Figure 3A). This demonstrates the value of droplet microfluidic HDX for capturing time-dependent differences in the uptake of highly dynamic peptides and protein regions. Measurements indicated faster kinetics than classical theory predicts^57^ (2.26 vs 0.80 Da at 10 ms), but this method may underestimate uptake.^58^ Our measurements more closely agreed with those produced using a high-velocity quenched flow apparatus^22^ and modern theoretical predictions^59,60^ (2.26 vs 2.34 Da at 10 ms) (Figure 3B).

**Figure 3:**
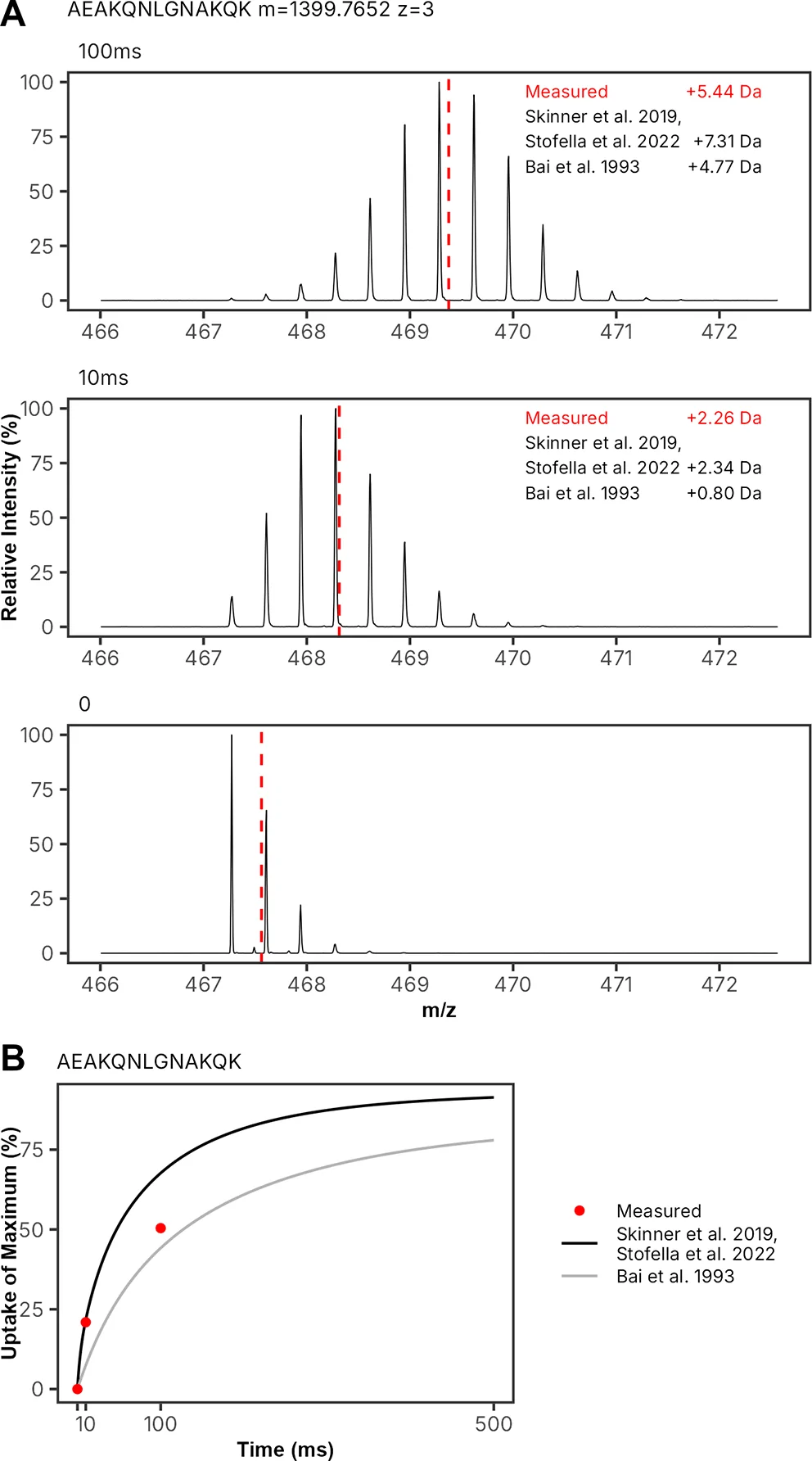
Millisecond HDX of peptides. (A) Stacked spectral plot of charge state +3 of peptide (AEAKQNLGNAKQK, [M + H]+ 1399.7652) at 0, 10, and 100 millisecond incubations from bottom to top. The red dashed line indicates the centroid, with measured and theoretical uptake values displayed as calculated using the methods described by Bai et al. 1993 and Skinner et al. 2019, Stofella et al. 2022. (B) Theoretical exchange of peptide (AEAKQNLGNAKQK) at pD 7.4 and 22 °C from 0 to 500 milliseconds. The red dots indicate the measured values at 10 and 100 milliseconds.

### Incubation Limit

We went on to determine the minimum possible incubation time point. Upon acid quenching, D_2_O exchange rates are reduced by 5–6 orders of magnitude but can nevertheless still proceed. This, so-called, forward exchange contribution can be negligible to the overall measured HDX with incubations of seconds to hours. However, at millisecond time scales where reduced incubations result in extremely low D_2_O uptake even low-level forward exchange can considerably contribute to relative mass gains. After D_2_O droplets merge with quench droplets, they are transported from the device to dry ice in 20 s, a time potentially sufficient for forward exchange to occur. It is therefore necessary to compare standard labelling with forward exchange to ensure that D_2_O uptake during 1, 10, and 100 millisecond HDX incubations does indeed originate from labelling and not just forward exchange post-quench. For standard labelling, we used the conventional sequence of D_2_O addition, incubation, and quench. In contrast, adding D_2_O at the quench step, not the initial step, allows forward exchange to be quantified.^32^ To aid comparison between forward exchange and standard labelling experiments, we worked with pre-digested CaM to provide putatively random-coil peptides.

Deuterium labeling is compared with forward exchange levels across the millisecond incubations (Figure 4A,B). With the 100 millisecond incubation, all peptides exhibit a higher degree of D_2_O labeling compared to forward exchange, demonstrating genuine labeling during incubation. However, shortening the incubations to 10 milliseconds shifts the labeling to forward exchange ratio. At 10 milliseconds, most peptides show a small degree of labeling relative to forward exchange. In place of CaM, more dynamic proteins such as intrinsically disordered proteins will have peptides with higher uptake levels at 10 milliseconds. To provide a fundamental definition of the limit of protein HDX-MS, the workflow can be reconfigured to prevent or at least greatly reduce forward exchange. For instance, substantially larger quench buffer proportions can be used, samples can be transferred to dry ice more rapidly (e.g., <1 s), or droplet HDX processing can be directly interfaced to MS by ESI.

**Figure 4:**
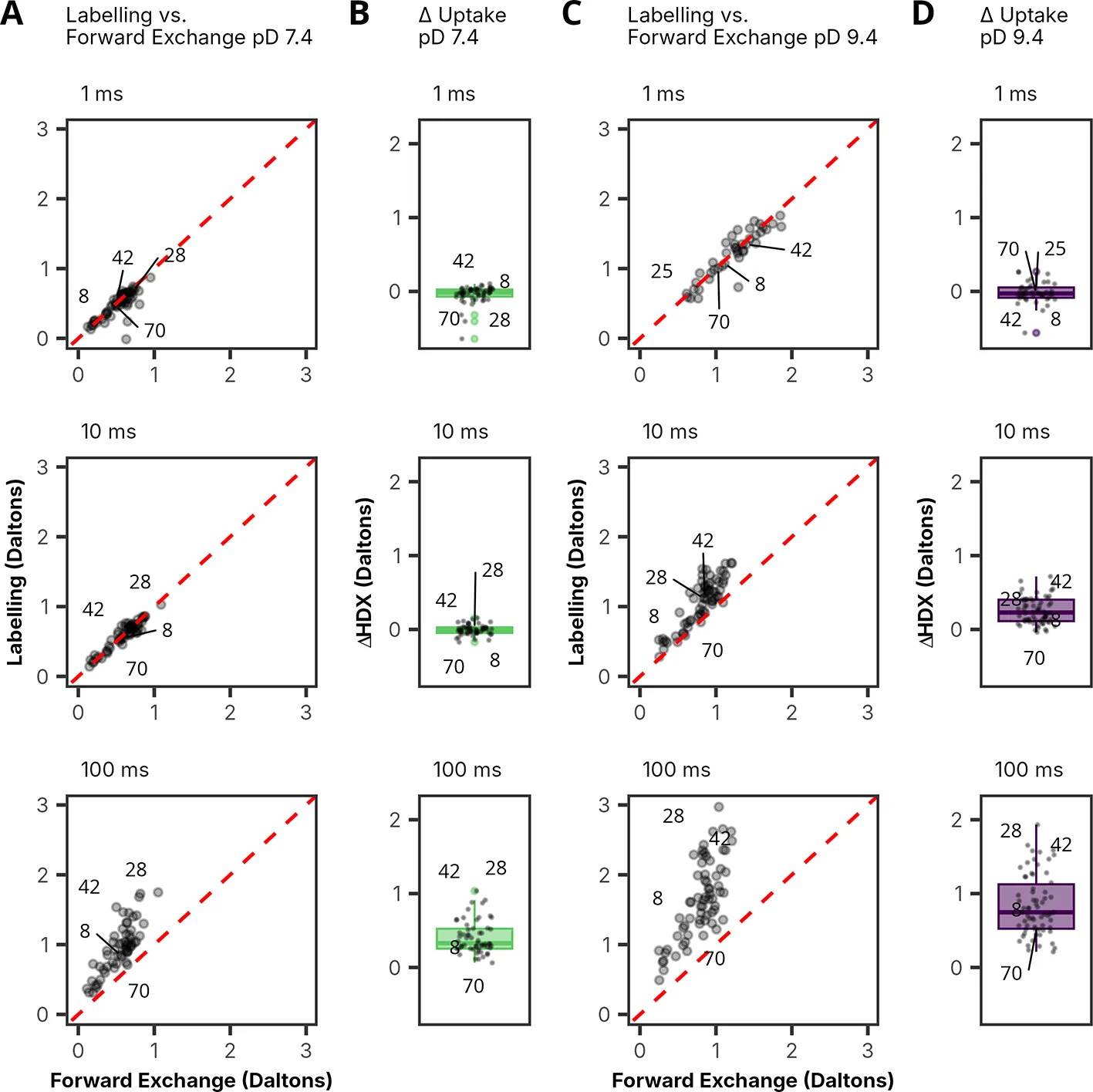
Time and pD-dependent labelling relative to forward exchange. (A,C) Parity plots of D_2_O labelling versus forward exchange at 1, 10, and 100 ms under pD 7.4 and pD 9.4 conditions. The red dashed line indicates parity of HDX between conditions and each point represents peptides identified in both conditions. Identifiers are shown for selected peptides. (B,D) Differential HDX labelling versus forward exchange of each CaM peptide at 1, 10, and 100 ms under pD 7.4 and pD 9.4 conditions.

The fluorescein switching microfluidics experiments demonstrate 1 millisecond incubations are feasible. However, at the 1 millisecond time-point, labelling and forward exchange levels are equally low at room temperature pD 7.4. This is also indicated by the low theoretical labelling levels calculated for a random coil incubated with D_2_O for 1 millisecond. Single millisecond labelling is therefore unrealistic at physiological pH and temperature. Since exchange kinetics increase with pH, we increased the pD (pH_read_ + 0.4) from 7.4 to 9.4 while retaining the quench condition at pH 2.5. As anticipated, higher D_2_O labelling was observed across the time course (Figure 4C,D). The low millisecond incubations may prove attractive for studying proteins with an alkaline optimal pH where labelling becomes saturated at conventional second time scales.^61,62^ To illustrate, theoretical D_2_O uptake is ∼100-fold higher for a 1 millisecond incubation at pD 9.4 than at pD 7.4. Taken together, we demonstrate that the lowest plausible HDX incubation for measuring amide backbone exchange in room temperature, pD 7.4 conditions is 10 milliseconds.

Temperature is another parameter affecting D_2_O uptake, with short incubation times having potential to be used for the investigation of thermostable proteins operating at high temperatures. In addition, the 1 millisecond HDX incubation may have merit for measuring D_2_O uptake on glycans, which show extremely fast proton exchange kinetics.^63^ Mindful of the variety of proteins and different HDX experiment requirements, we evaluated whether reliable droplet processing was sensitive to temperature extremes and commonplace buffer components that may influence viscosity and surface tension. Droplet generation and merging was unaffected by temperatures ranging from 6 °C to 42 °C, and insensitive to the inclusion of dimethyl sulfoxide (DMSO, 5%), glycerol (5%), the detergent dodecyl maltoside (0.05%), and the denaturant urea (6M). While far from exhaustive, this experimental screen indicates the general applicability of droplet HDX for probing fast exchanging protein regions. In the case of membrane proteins or membrane mimetics, compatibility with the fluoro-oil carrier will need to be assessed.

### Millisecond HDX Investigation of Calmodulin Dynamics

Next, we sought to investigate CaM protein dynamics in Ca^2+^-loaded and Ca^2+^-unloaded states (holo and apo, respectively) to demonstrate measurement of early exchange processes. CaM is a small, 148 amino acid, globular protein that has been extensively studied using various biophysical techniques.^64–68^ Calmodulin has two globular domains, the N- and C-lobes which are connected via a flexible linker to form a dumbbell structure.^69,70^ Both lobes each contain two EF-hand motif Ca^2+^ binding sites,^71^ and upon Ca^2+^ binding, the linker domain becomes more flexible, enabling the N- and C- globular domains to come together in a scissor-like action.^72–74^ The well-known structure and HDX-MS profiles make it a model for protein dynamics and a potential good test case for HDX technology development.^75^

Droplet microfluidic HDX-MS analysis was performed at 10 and 100 milliseconds and integrated with longer deuteration time points, 10, 100, and 1000 s, collected through manual HDX labelling. The coordination of Ca^2+^ is expected to provide stabilization of the binding site, causing the formation of a less dynamic structure. Characteristically, Ca^2+^-binding caused backbone stabilization across many parts of the protein, most dramatically within the C-domain lobe, and no measurable change within the region covering residues 35–51 within the N-domain lobe (Figures 5A, C–E and S3). Whereas Ca^2+^-loading caused destabilization within the linker region, including peptides spanning residues 63–84 at early (10 s) time points). This effect vanished at later time points and even crossed over as shown for peptide 63-69 (Figure 5A,D,E). As expected, significant differences between the apo and holo states were observed at longer deuteration time points, confirming the effectiveness of the Ca^2+^-stripping procedure used to generate apo CaM from bovine testis (Cat. No. P1431) prior to HDX-MS analysis (Figure 5A; EF3 and EF4 peptides). In contrast, application of the same Ca^2+^-stripping procedure to recombinant bovine calmodulin expressed in *E. coli* (Cat. No. C4874-1MG, Merck) failed to generate a fully apo state, resulting in HDX profiles that differed only marginally from those of the Ca^2+^-loaded holo state (Supporting Information Figure S4).

**Figure 5:**
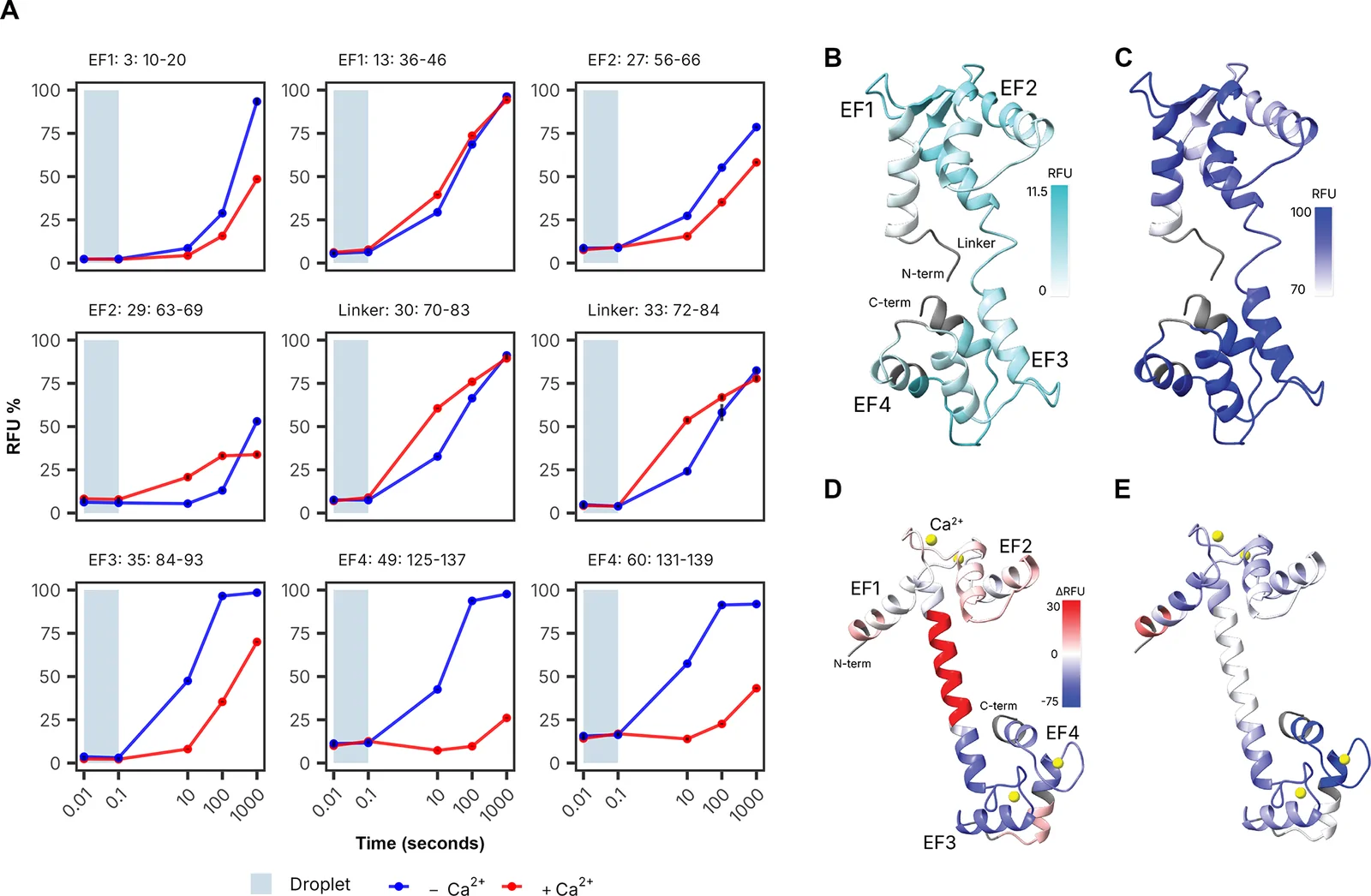
Calmodulin dynamics. (A) Time-dependent D_2_O uptake plots for peptide areas of interest spanning N-lobe, linker, and C-lobe. Red and blue traces show measurements with and without Ca^2+^, respectively. 10 and 100 millisecond incubations were processed using droplet microfluidics (light blue panel) and the 10, 100, and 1000 s incubations were processed manually. Error bars (*n* = 3) typically too small to be observed. All other uptake plots are presented in Figure S3. Structures (1cfd.pdb) in (B,C) show the relative fractional uptake of apo CaM at 10 milliseconds and 1000 s, respectively. A light blue gradient ranging from 0 to 11.5% RFU was applied to the 10 millisecond structure, and a dark blue gradient ranging from 70 to 100% RFU was applied to the 1000 s structure. Structures (1cll.pdb) in (D,E) show the differential uptake between apo and holo CaM (calculated as holo-apo) at 10 and 1000 s, respectively. Increase and decrease in protein dynamics upon Ca^2+^-binding are highlighted in red and blue, respectively. Areas without peptide coverage are gray.

Millisecond-time scale HDX primarily reports on intrinsically disordered or weakly structured regions due to their rapid backbone accessibility, whereas longer labelling times probe broader structural dynamics. Extending HDX into the millisecond regime revealed no major differences between Ca^2+^-bound and Ca^2+^-free states, indicating that the protein remains stably folded in both conditions. HDX at the theoretical lower limit (∼10 ms) establishes a structural baseline from which regions of differential stability can be resolved. At 10 milliseconds, mapping relative fractional deuteration onto the structure revealed distinct segments with substantial early exchange (>10% RFU) and others without exchange (Figure 5B). The EF-hand regions of the N- and C-lobe (EF1-1 and EF3-4, respectively) displayed rapidly exchanging regions, reflecting increased structural plasticity. Such intrinsic flexibility in the Ca^2+^-binding regions is a defining feature of signaling protein domains that typically participate in high-affinity, low-specificity interactions.^76,77^ This is consistent with molecular dynamics studies indicating that the conformational plasticity of CaM Ca^2+^-binding domains gives rise to a heterogeneous ensemble of folded substates, each potentially exposing distinct surface topologies that may facilitate the binding of a broad spectrum of target proteins.^78,79^ Supporting these interpretations, millisecond HDX identifies regions with lower structural stability even within stably folded structures.^80^ Contrary to the heightened plasticity in the EF-hands of the N- and C-lobes, the flexible linker—which is not involved in Ca^2+^ sensing but instead allows diverse conformations to be adopted^81^—only showed increased plasticity upon Ca^2+^-binding, but not at millisecond time points (Figure 5A,D). Although the N- and C-lobes of calmodulin share similar EF-hand architectures, they function as distinct Ca^2+^ sensors. The C-lobe shows higher affinity and slower Ca^2+^ dissociation, providing a more sustained response, whereas the N-lobe exhibits lower affinity but faster dissociation kinetics, enabling rapid detection of transient Ca^2+^ elevations. These established functional asymmetries are indicated by HDX at conventional time points, where a higher degree of stabilization in the C-lobe relative to the N-lobe, is observed by HDX-MS.^82^ Such distinction cannot be made at millisecond time scales, yet such short time scales are able to identify fast-paced backbone exchange-competent areas associated with Ca^2+^ sensing.

Calmodulin was chosen as a well-known and commercially available shape change model, making it a potential candidate for benchmarking the droplet microfluidic HDX analysis method. However, calmodulin proved sub-optimal for demonstrating the benefits of millisecond HDX. Other benchmarking experiments have used short peptides with high uptake rates to explore millisecond D_2_O labeling.^22^ These peptide models provide a good reference basis for the deuterium exchange of highly dynamic proteins and intrinsically disordered protein regions. In summary, we recognize millisecond HDX is a nascent field and anticipate the droplet microfluidics HDX method offers good opportunity for biological discovery.

## Conclusions

We present a droplet microfluidic HDX approach for rapidly mixing proteins with D_2_O, uniform incubations in the millisecond regime, and droplet merging for rapid acid quenching. Robust droplet merging was achieved by using surfactant-free oil in combination with expansion channel elements for the synchronization and contacting of droplet pairs. The high diffusive flux of D_2_O and protons allows microsecond mixing, making incubations as short as 1 millisecond feasible. We show that forward exchange becomes apparent at millisecond time scales and that D_2_O labeling is the rate-limiting step, indicating a 10 millisecond base-rate as the shortest plausible labeling time for typical physiological conditions. Droplet HDX combined with conventional HDX was used to investigate calmodulin dynamics, with substantial millisecond uptake (>5% RFU) within stably folded protein domains (EF-hand motifs) that are crucial for Ca^2+^ binding. These decoupled droplet microfluidic HDX demonstrations provide proof of principle for other lines of research investigating the dynamics of intrinsically disordered proteins, highly dynamic protein regions, and conformation switching. Droplet microfluidic HDX is inexpensive and requires minimal sample, critical features that enable broad user adoption. The technology can be further advanced by integration with downstream microfluidic devices for protein digestion, chromatographic separation, and electrospray ionization for direct coupling with MS^83^ to aid automation and reduce both forward and back exchange. Beyond this, we can envisage developments in both microfluidics and HDX-MS allowing protein dynamics captured within single droplets or even within single cells (i.e., proteome plasticity) to be studied.

## Supplementary Material











## Notes

Bovine calmodulin uptake plots Figure S4. *E. coli* calmodulin uptake plots Table S1 (ZIP)
Figure S1. Droplet HDX concept Figure S2. Droplet merging positions Figure S3 (PDF)
HDX Summary Video S1: High-speed microscopy recording of the “chop and exchange” method (ZIP)
Video S2: High-speed microscopy recording of the “drag and merge” method (ZIP)
Video S3: High-speed microscopy recording of the “collide and merge” method (ZIP)
