# Supplementary material for: Droplet Microfluidic Hydrogen/Deuterium Exchange for Investigating Protein Dynamics with Millisecond Precision
Source: Anal Chem. 2026 Sep 28;98(40):29216–27. doi: 10.1021/acs.analchem.6c02766 (PMC13670895; doi:10.1021/acs.analchem.6c02766)
Supplement: Supplementary file 1 [file ac6c02766_si_002.pdf]

Supporting Information

Droplet microfluidic hydrogen/deuterium exchange for investigating protein dynamics with millisecond precision

Dietmar Hammerschmid[a*†], Jakub Sys[a†], Alistair Bailey[b,d], Simon I.R. Lane[c,d], Max Saito[b], Theo Hornsey[a], Niall Hanrahan[c,d], Andy van Hateren[b,d], Howard Broughton[e], Alfonso Espada[e], Eamonn Reading[a,d] and Jonathan West[b,d*]

[a] School of Biological Sciences, Faculty of Environmental and Life Sciences, University of Southampton, SO17 1BJ, UK

[b] Cancer Sciences, Faculty of Medicine, University of Southampton, SO17 1BJ, UK

[c] School of Chemistry, Faculty of Engineering and Physical Sciences, University of Southampton, SO17 1BJ, UK

[d] Institute for Life Sciences, University of Southampton, SO17 1BJ, UK

[e] Centro de Investigación Lilly, SA, Avenida de la Industria 30, 28108, Alcobendas, Spain

† equal contributions

* corresponding authors: dietmar.hammerschmid@biophys.mpg.de; J.J.West@soton.ac.uk

Table of Contents

Figure S1. Droplet HDX concept page S2

Figure S2. Droplet merging positions page S2

Figure S3. Bovine calmodulin uptake plots page S3

Figure S4. *Escherichia coli* calmodulin uptake plots page S4

Table S1: HDX Summary page S5

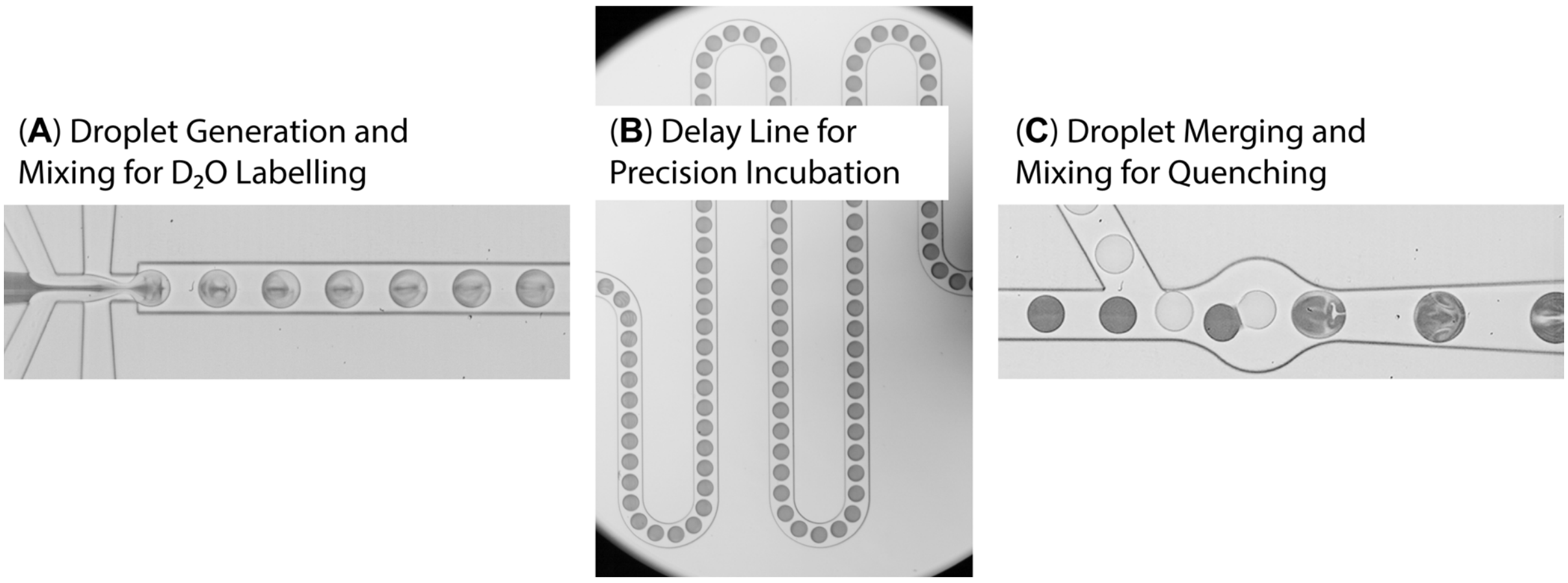

Figure S1. Concept for fast HDX using droplet microfluidics. Protein is rapidly mixed with D_2O during droplet generation and transport (**A**), single-file droplet transport ensures uniform incubation times (**B**), and droplets collide and merge in an expansion-contraction element for rapid acid quenching (**C**).

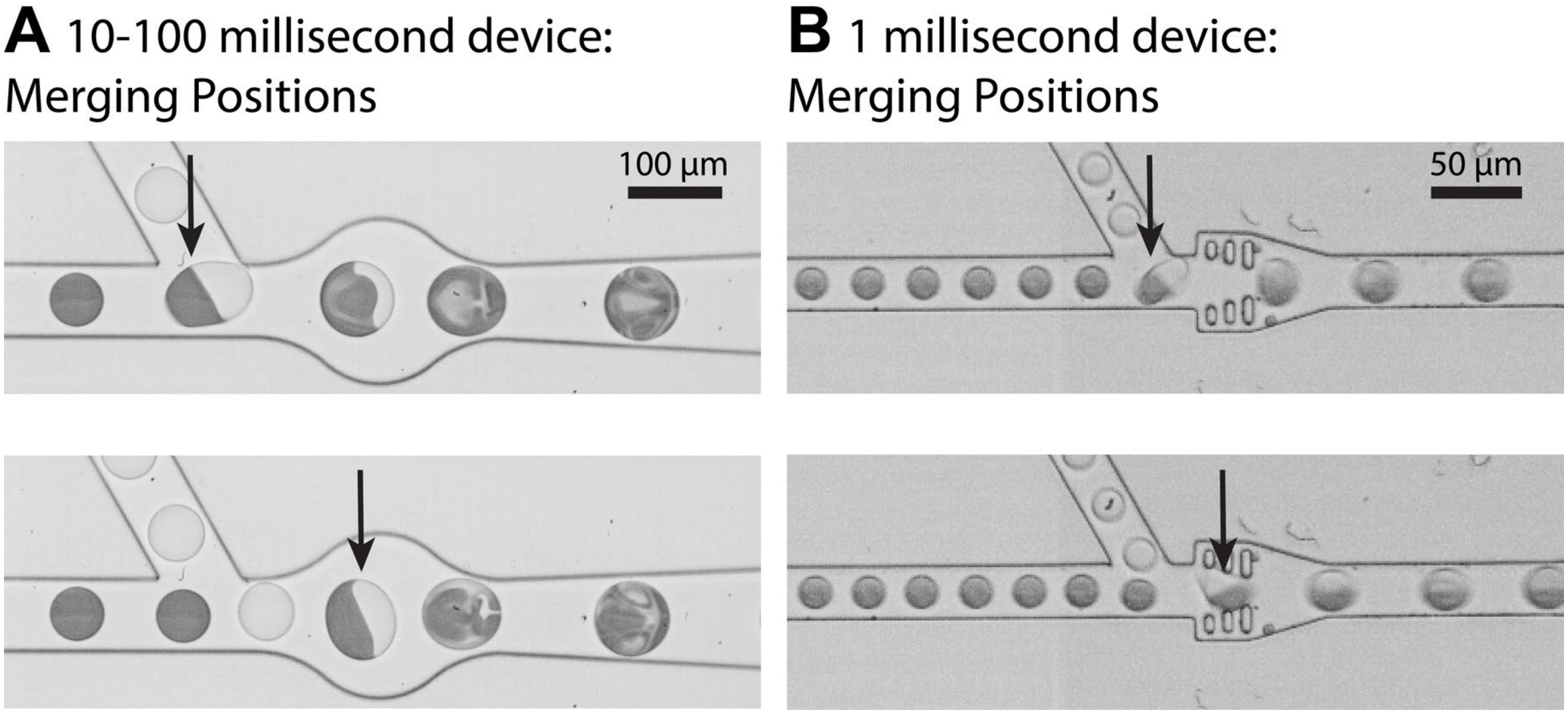

Figure S2. Merging occurs where channels converge (**A,B**) and immediately downstream at the channel expansion-contraction element in the 10 and 100 milliseconds incubation devices (**A**) or between the oil drainage pillar structures in the 1 millisecond incubation device (**B**). Arrows indicate merging positions.

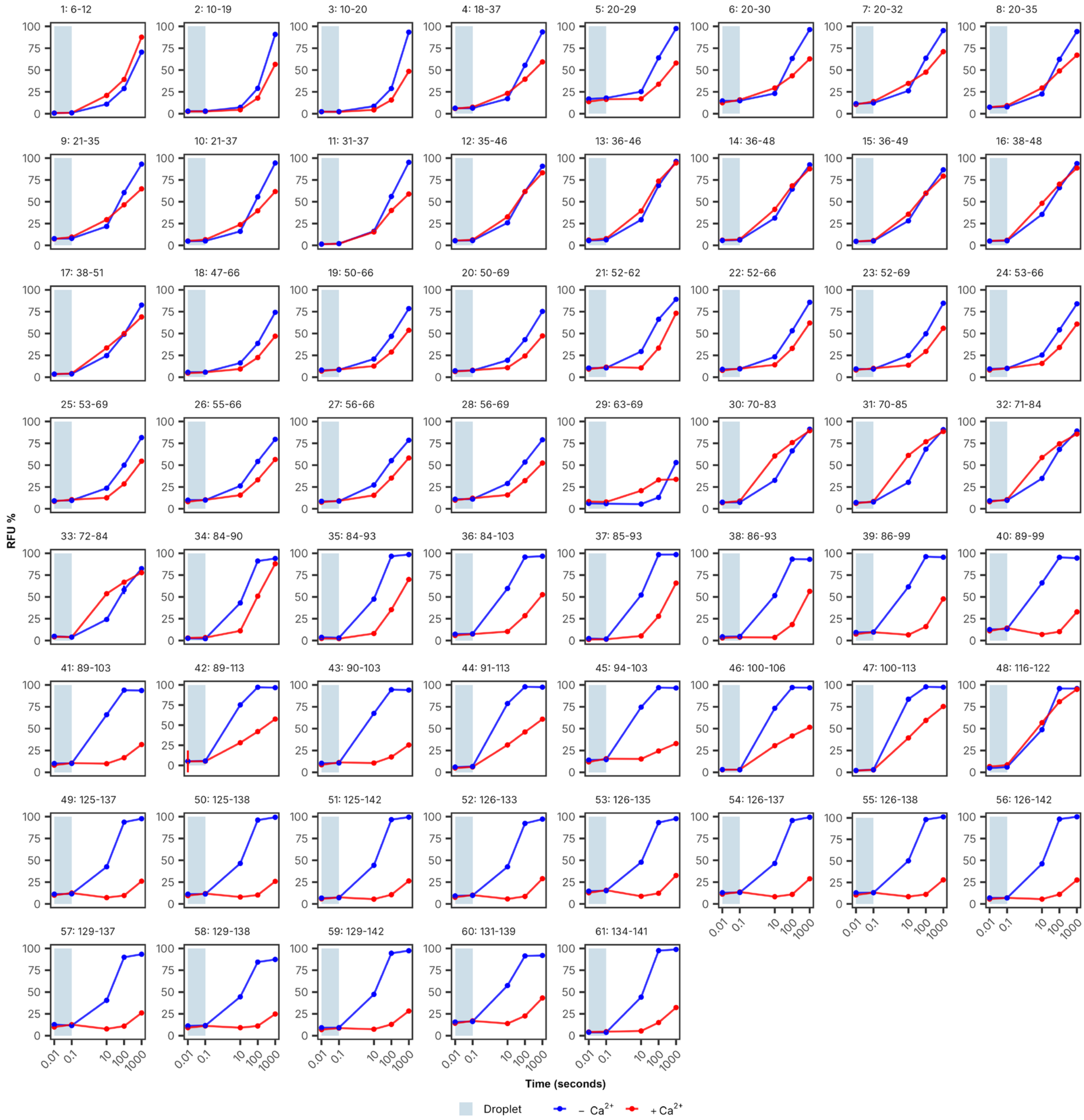

Figure S3. Time-dependent D_2O uptake plots for all calmodulin peptides sourced from bovine testes with and without Ca^{2+}. The 10 and 100 millisecond incubations were achieved using droplet microfluidics (shaded blue) and the 10, 100 and 1000 second incubations by manual handling.

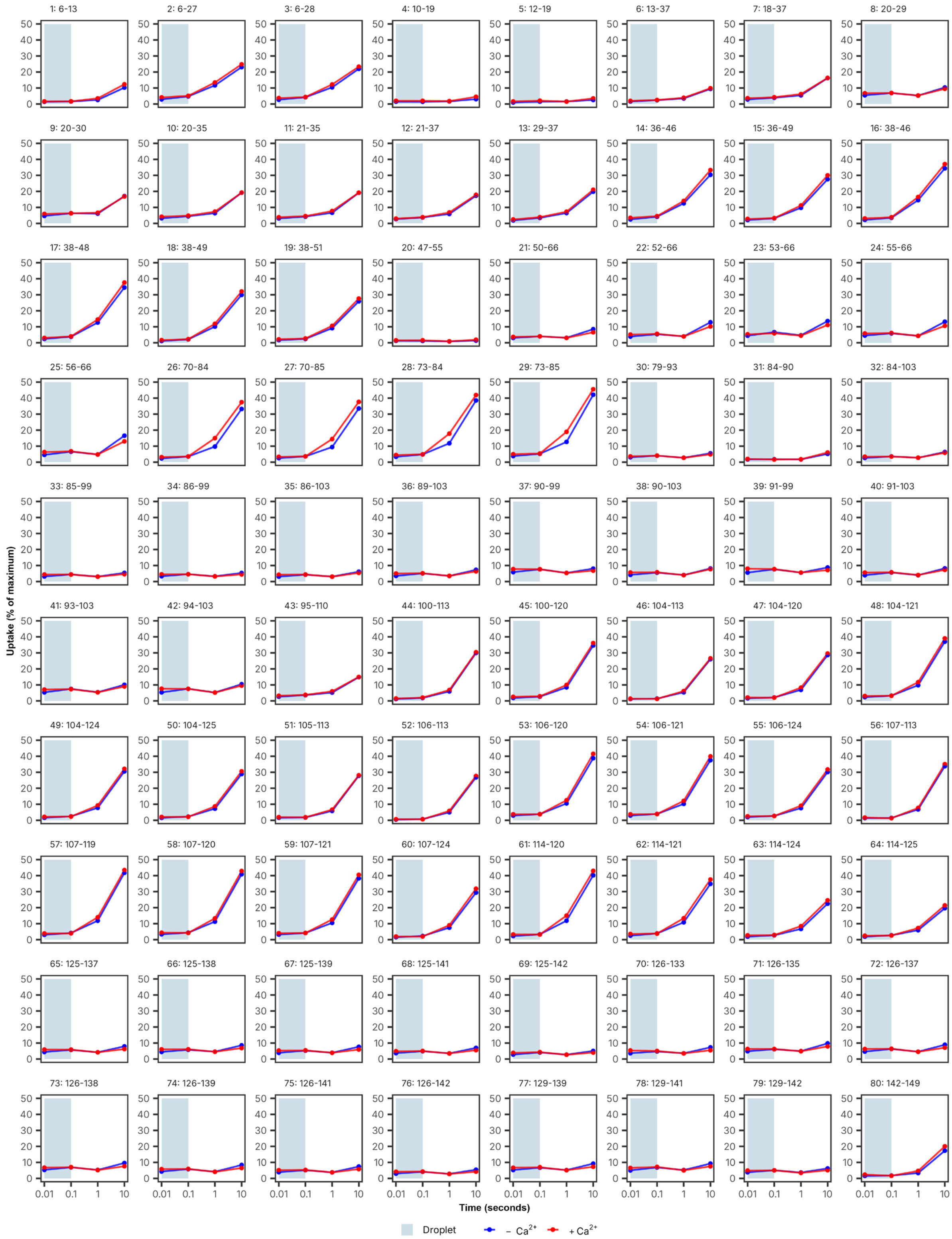

Figure S4. Time-dependent D_2O uptake plots for all recombinant calmodulin peptides sourced from *Escherichia coli* with and without Ca^{2+}. The 10 and 100 millisecond incubations were achieved using droplet microfluidics (shaded blue) and the 1 and 10 second incubations by manual handling.

Table S1: HDX Summary

HDX reaction details	20 mM HEPES, pH 7.4 (pD 7.0) → apo<br>20 mM HEPES + 1 mM $CaCl_2$, pH 7.4 (pD 7.0) → holo
HDX time course (sec)	0.01, 0.1 (microfluidics)<br>10, 100, 1000 (manual labelling)
HDX control samples	5 (no labelling), 3 (fully deuterated control (Dmax))
* Back-exchange (mean / IQR)	32.02% ± 0.26%
# of Peptides	61
Sequence coverage	89 %
Average peptide length /<br>Redundancy	13<br>5.97
Replicates	3 technical
Repeatability	microfluidics: 0.049 Da (average standard deviation)<br>manual labelling: 0.033 Da (average standard deviation)
Significant differences in HDX	HDX > 0.5 Da and p-value < 0.01 in Welch's t-test

* Back exchange of the HDX-MS setup has been determined in *Chromatographic Phospholipid Trapping for Automated H/D Exchange Mass Spectrometry of Membrane Protein-Lipid Assemblies. Dietmar Hammerschmid, Valeria Calvaresi, Chloe Bailey, Benjamin Russell Lewis, Argyris Politis, Michael Morris, Laetitia Denbigh, Malcolm Anderson, and Eamonn Reading. Analytical Chemistry 2023 95 (5), 3002-3011. DOI: 10.1021/acs.analchem.2c04876*
